# Mouse Heart Rate in a Human: Diagnostic Mystery of an Extreme Tachyarrhythmia

**DOI:** 10.1016/s0972-6292(16)30463-6

**Published:** 2012-01-31

**Authors:** Lovely Chhabra, Narender Goel, Laxman Prajapat, David H Spodick, Sanjeev Goyal

**Affiliations:** 1 Department of Internal Medicine, Saint Vincent Hospital, University of Massachusetts Medical School, Worcester, Massachusetts; 2 Department of Cardiovascular Medicine Saint Vincent Hospital, University of Massachusetts Medical School, Worcester, Massachusetts; 3 Department of Electrophysiology, Saint Vincent Hospital, University of Massachusetts Medical School, Worcester, Massachusetts

**Keywords:** Tachyarrhythmia, Atrial flutter, Atrial fibrillation, Quadriplegia

## Abstract

We report telemetry recording of an extreme non-fatal tachyarrhythmia noted in a hospitalized quadriplegic male with history of atrial fibrillation where the average ventricular conduction rate was found to be about 600 beats per minute and was associated with transient syncope. A medical literature review suggests that the fastest human ventricular conduction rate reported to date in a tachyarrhythmia is 480 beats per minute. We therefore report the fastest human heart rate noted in a tachyarrhythmia and the most probable mechanism of this arrhythmia being a rapid atrial fibrillation with 1:1 conduction in the setting of probable co-existing multiple bypass tracts.

## Introduction

The maximum human heart rate conduction is primarily limited by the absolute refractory period (ARP) of the AV junction which theoretically limits the maximum conduction rate to about 300 beats per minute. However there have been several cases in the literature which have reported the heart rates of above 300 per minute. The fastest human ventricular conduction rate reported to date is a conducted tachyarrhythmia with ventricular rate of 480 beats per minute. We report telemetry recording of an extreme non-fatal tachyarrhythmia noted in a hospitalized quadriplegic male with history of atrial fibrillation where the average ventricular rate was about 600 beats per minute and was associated with a transient syncope.

## Case Presentation

**Figure 1:**
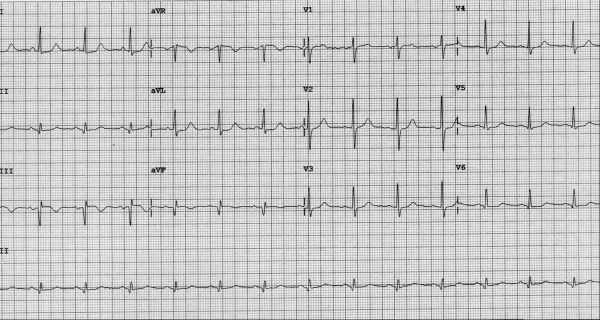
Electrocardiogram at the time of admission revealed a normal sinus rhythm with subtle ST elevations and deep Q waves in inferior leads.

**Figure 2:**
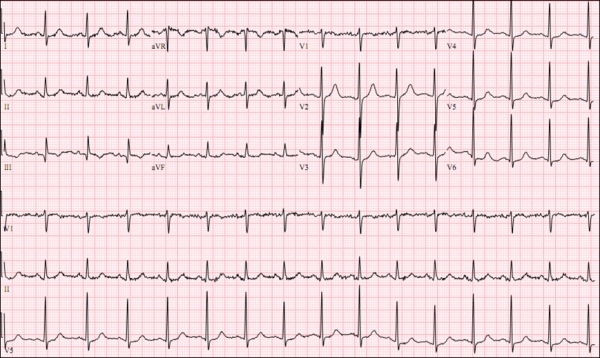
Old electrocardiogram for comparison.

**Figure 3:**
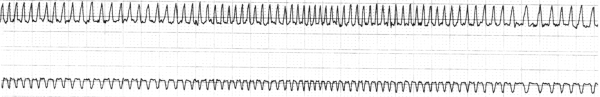
Full telemetry strip revealing the extreme tachyarrhythmia with an average ventricular rate of about 600 beats per minute. Patient experienced transient syncopy during this event.

A 57 year old quadriplegic male was brought to the hospital with complaints of presyncope, fall from his wheelchair and ongoing substernal chest pressure of a few hours duration. Past medical history included Non-ST elevation myocardial infarction (NSTEMI) with a drug eluting stent (DES) placed in the right coronary artery (RCA) three years ago, paroxysmal atrial fibrillation and quadriplegia secondary to trauma in a motor vehicular accident ten years ago. He was a non-smoker and a resident of a long term nursing care facility. His home medications included aspirin, metoprolol, simvastatin and warfarin. Initial vital signs were stable. Physical examination was unremarkable except for quadriplegia. Initial laboratory data revealed a normal hemogram and basic metabolic panel but an elevated troponin value of 0.2 ng/mL. Electrocardiogram (ECG) at admission (Figure 1) revealed normal sinus rhythm with subtle ST elevations and deep Q waves in inferior leads which were new changes as compared to his previous ECG (Figure 2). He was initially started on optimal medical management for acute coronary syndrome including nitrates and heparin. A cardiac catheterization was initially deferred per patient's wishes and in view of his chronic medical comorbidities. He was free of his cardiac symptoms the following day of his admission and the follow-up troponin value was < 0.1 ng/mL. On Day 3, he experienced recurrent chest pains and telemetry revealed multiple episodes of rapid non-sustained ventricular arrhythmias and a few episodes of rapid paroxysmal atrial fibrillation. One of the episodes of severe chest pain was followed by transient syncopy and a tachyarrhythmia (noticed on telemetry) lasting for about 20 seconds with a ventricular rate of about 600 beats per minute, which gradually slowed over a few seconds to a rate of 300 beats per minute before spontaneous conversion to normal sinus rhythm (Figure 3). Follow-up troponin value was 1 ng/mL. After an extensive discussion with the patient and his family, he agreed to an urgent cardiac catheterization which revealed complete occlusion of his distal RCA which was successfully revascularized followed by DES placement. An electrophysiological (EP) study was recommended but the patient and his family declined any further diagnostic study or intervention. He remained clinically stable and was discharged two days post coronary intervention without any further noticed significant tele-alarm activity.

## Discussion

The unique presentation of this tachyarrhythmia in our patient was a diagnostic challenge for the expert panel of our cardiologists and electrophysiologists. In the absence of availability of an electrophysiological study and a 12-lead capture of this arrhythmic event, we believe that the most likely mechanism of this arrhythmia could be atrial fibrillation with 1:1 conduction in the probable setting of co-existent multiple bypass tracts considering presence of possible fibrillatory waves, the irregular nature of the rhythm during its slow phase and the patient's past history of paroxysmal atrial fibrillation. Without a 12 lead capture and an EP study, one could argue a possible differential diagnosis of a teleartifact (most likely a paper-speed artifact) however it is excluded because the paper speed was set at the regular standard (25 mm/s) and the telealarm morphology on the monitor was consistent with the one on the recorded telestrip excluding the possibility of a recording speed error. The true nature of this tachyarrhythmia is also suggested by the obvious co-existing clinical facts not otherwise explained by an artifact and these include the patient's symptomatology (chest pain followed by a transient syncopy during the event), non-artifactual nature of the QRS morphology, elevation of the downward trending troponin value (most likely induced by a demand-supply mismatch from this extreme tachyarrhythmia in the setting of an underlying occlusive CAD), and confirmed functional appropriateness of the telemetry monitor. The wide QRS morphology during the slow phase of the tachyarrhythmia could be possibly related to a deceleration-related aberrancy [1,2].

With normal cardiac physiology, it is known that following the action potential (AP), the absolute refractory period (ARP) prevents another AP until the channels are reset at the AV Junction. The ARP of the AV Node lasts about 0.2 seconds limiting the heart rate to 300/min in theory. Following the ARP is the relative refractory period (RRP), and the stimulus needs to be greater than before, as the original electrical potential across the membrane hasn't been fully restored. So, 300 beats per minute is not sustainable for long, as the stimulus needs to be progressively greater each time to generate the next AP. Another possible factor is the cardiac myocyte action potential duration which is normally about 200 msec which again theoretically would limit the heart rate to about 300 beats per minute. Heart rate conduction above 300 beats per minute would thus involve the presence of at least a bypass tract, shorter cardiac myocyte AP duration and also probable selective cardiac myocyte activation. A literature search has revealed several reports of extreme tachycardia. In 1943, Joseph Edeikein reported two cases; first case was of a 47 year-old female with myocardial ischemia who experienced two separate non-fatal paroxysms of supraventricular arrhythmia; one with a ventricular rate of 310 beats per minute lasting for 12 hours and second with a ventricular rate of 303 beats per minute lasting for 34.5 hours. The second case he reported was an infant aged 22 days that died after two days of sustained tachycardia with conducted ventricular rates reported above 300 beats per minute [3]. Smelin et al reported a case of spontaneous atrial flutter in an adult with 1:1 conduction at rate of 300 beats per minute [4]. In 1949, Silverman et al reported a fatal case of supraventricular tachycardia in an infant with a rate of 365 beats per minute [5]. Lisowski et al studied atrial flutter in perinatal age group and the effect of maternally administered antiarrhythmic agents and they had reported a fetus with fatal rapid 1:1 atrioventricular conduction at 480 beats per minute which was probably the fastest human heart rate reported to date in the standard medical literature [6]. A non-medical literature search has also revealed a case report of a Danish audiologist, Ole Bentzen, who died laughing while watching the movie "A Fish Called Wanda" in 1989. His heart rate was reported between 250 and 500 beats per minute, before he succumbed to cardiac arrest [7]. Our case thus describes the fastest human heart rate ever reported. The patient spontaneously converted back to normal sinus rhythm after about 20 seconds and didn't require cardioversion. Could an autonomic dysregulation (with history of his quadriplegia) in the setting of possible underlying concealed multiple bypass tracts have played a role in precipitating this extreme tachyarrhythmia remains a question of debate?

## References

[R1] Wagner GS (2001). Marriott's Practical Electrocardiology, 10th Ed.

[R2] Mussumi RA (1968). Bradycardia-dependent bundle branch block. A critique and proposed criteria. Circulation.

[R3] Edeiken J (1943). Extreme Tachycardia: With Report of Non-Fatal Paroxysms Following Myocardial Infarction. American Journal of the Medical Sciences.

[R4] Smelin A (1953). Tachycardia and spontaneous flutter in an adult; report of a case with rate of 300 and 1:1 flutter. AMA Arch Intern Med.

[R5] Silverman JJ (1949). Paroxysmal tachycardia with a ventricular rate of 365 per minute. Am Heart J.

[R6] Lisowski LA (2000). Atrial flutter in the perinatal age group: diagnosis, management and outcome. J Am Coll Cardiol.

[R7] Wallechinsky D (2008). The Book Of Lists: Death: 9 People Who Died Laughing.

